# Improving Self-management of Type 2 Diabetes in Latinx Patients: Protocol for a Sequential Multiple Assignment Randomized Trial Involving Community Health Workers, Registered Nurses, and Family Members

**DOI:** 10.2196/44793

**Published:** 2023-01-16

**Authors:** Alex Kopelowicz, Karabi Nandy, Maria Elena Ruiz, Rhonda Polzin, Kevin Kurator, Soma Wali

**Affiliations:** 1 Department of Psychiatry & Biobehavioral Sciences David Geffen School of Medicine at UCLA Los Angeles, CA United States; 2 Department of Population & Data Sciences University of Texas Southwestern Medical Center Dallas, TX United States; 3 University of California, Los Angeles School of Nursing Los Angeles, CA United States; 4 Department of Nursing Olive View-UCLA Medical Center Sylmar, CA United States; 5 Department of Medicine Olive View-UCLA Medical Center Sylmar, CA United States

**Keywords:** community health workers, diabetes education, Latinx, nurses, promotores, type 2 diabetes

## Abstract

**Background:**

The rate of Type 2 diabetes mellitus (T2DM) among Mexican American individuals is 16.3%, about twice that of non-Hispanic White individuals. While a number of education approaches have been developed and shown to improve diabetes self-management behaviors and glycemic control for Spanish-speaking Latinx patients with T2DM, there is little research to guide health practitioners regarding which interventions to apply and when so that resources are used efficiently, and treatment outcomes are maximized.

**Objective:**

This study aimed to describe an adaptive intervention that integrates community mental health workers, diabetes nurse educators, family members, and patients as partners in care while promoting diabetes self-management for Mexican American individuals with T2DM. The project incorporates four evidence-based, culturally tailored treatments to determine what sequence of intervention strategies work most efficiently and for whom. Given the increasing prevalence of T2DM, achieving better control of diabetes and lowering the associated medical complications experienced disproportionally by Mexican American individuals is a public health priority.

**Methods:**

Funded by the National Institute of Nursing Research (National Institutes of Health grant R01 NR015809), this project used a sequential multiple assignment randomized trial and included 330 Spanish-speaking Latinx patients with T2DM. In the first phase of the study, subjects were randomly assigned to an evidence-based diabetes self-management educational program called Tomando Control delivered in a group format for 6, biweekly 1.5-hour sessions, led either by a community health worker or a diabetes nurse educator. In the second phase of the study, those subjects who did not improve their diabetes self-management behaviors were rerandomized to receive either an augmented version of Tomando Control or a multifamily group treatment focused on problem-solving. The primary outcome measure was the “Summary of Diabetes Self-Care Activities.” Evaluations were made at baseline and at 3, 6, and 12 months.

**Results:**

This study was funded in June 2016 for a period of 5 years. Institutional review board approval was obtained in November 2016. Between March 2017 and September 2020, a total of 330 patients were recruited from the outpatient primary care clinics of Olive View-UCLA Medical Center, with a brief hiatus between May 2020 and July 2020 due to COVID-19 restrictions. The study interventions were completed in December 2020. Data collection began in March 2017 and was completed in December 2021. Data analysis is expected to be completed in Spring 2023, and results will be published in Fall 2023.

**Conclusions:**

The results of this trial should help practitioners in selecting the optimal approach for improving diabetes self-management in Spanish-speaking, Latinx patients with T2DM.

**Trial Registration:**

ClinicalTrials.gov NCT03092063; https://clinicaltrials.gov/ct2/show/NCT03092063

**International Registered Report Identifier (IRRID):**

DERR1-10.2196/44793

## Introduction

### Background and Rationale

At 53 million people, the Latinx population is the largest minority group in the United States [1]. More than half of these are of Mexican origin, and this group has the lowest median age of any racial or ethnic group in the United States. Despite this, Latinx people living in the United States exhibit a disproportionate burden from type 2 diabetes mellitus (T2DM), with about double the prevalence among non-Hispanic White individuals [2]. A recent review of studies demonstrating an increased prevalence of diabetes and its complications among Latinx individuals compared with non-Hispanic White individuals suggested that low health literacy, lack of access to health care resources, and language barriers are among the main causes of T2DM ethnic disparities in the United States [3].

Over the past 20 years, several diabetes self-management education (DSME) approaches have been developed and shown to improve diabetes self-management behaviors and glycemic control as well as other health outcomes for Mexican American individuals with T2DM [4-9]. Numerous approaches have been developed, many of which share common elements including community involvement, face-to-face encounters, ongoing participation of a multidisciplinary treatment team, targeting the development of skills to promote behavior change, the provision of prompts and reminders, the use of a prior needs assessment to inform intervention design, and an explicit focus on social-contextual issues [10,11]. While each of these evidence-based DSME approaches has demonstrated advantages over usual practices in Mexican American communities, these interventions differ in their format (individual vs group), the type of provider used to deliver the DSME intervention (health professional vs lay community health worker or, in this study, *promotore*), and treatment target (patient vs patient and family). Such differences could be impactful both in terms of overall patient outcomes as well as economic feasibility for health care systems considering their implementation; however, there is little research to date to guide health practitioners regarding which interventions to apply and when in order to maximize treatment outcomes and resource efficiency.

An additional gap in the literature stems from the finding that fewer than 40% of subjects offered these treatments achieved levels of hemoglobin A1c (HbA1c) in accordance with the standards established by the American Diabetes Association [12]. Identifying nonresponding patients early in the treatment process and implementing more culturally appropriate, family-centered approaches could forestall long-term adverse consequences. The rationale of this study is to fill this gap of knowledge by comparing 4 different approaches to enhance diabetes self-management of Hispanic patients with T2DM.

### Registered Nurses Versus Community Health Workers

Many educational and management strategies developed and implemented to address the specific needs of the Mexican American population were originally designed to involve registered nurses (RNs), certified diabetes educators, or dietitians as the primary patient educators [6,8,9]. The limited availability of such specialized providers in general, and bilingual or bicultural providers, in particular, prompted many interventions to use *promotores* to handle group educational and skill reinforcement sessions as adjuncts to the primary educators. In some programs, the role of the *promotore* was modified such that the *promotore* became the principal diabetes health educator, thus replacing highly educated personnel such as certified nurses for patient education and long-term case management [13-19].

The evidence regarding the efficacy of *promotore*-delivered interventions is mixed and incomplete. Some studies have found that interventions with community health workers do indeed have an impact when compared to usual clinical practice [8,9,16-18,20]. However, it has also been suggested that this may not be the case [19], and at least one study using focus groups with primarily Mexican American patients found that these patients strongly preferred trained health care professionals like nurses [12]. To date, there has been no study that has directly compared similar DSME interventions delivered by a community health worker (ie, a *promotore*) versus a health care professional such as a nurse.

### Family Incorporation

Social support has been recognized as a crucial component of health and behavior change, and family members are the most significant source of that support [21]. Family behaviors and attitudes can support or challenge a patient’s adaptation to illness and subsequently a patient’s confidence, intent, and willingness to implement disease-management strategies [22], and a recent meta-analysis has suggested that addressing family functioning and problem-solving in addition to teaching diabetes self-management skills is critical to success [23]. Mexican American individuals with T2DM have cited attitudes, perceptions, and preferences of family members as significant barriers to making recommended changes in their diet and exercise patterns [24]. Interventions that target familial support to improve diabetes self-management have been effective within the Mexican American population [25]. Despite this evidence, most of the abovementioned interventions designed and tested for Spanish-speaking Hispanic individuals only encourage the involvement of families in DSME interventions; none explicitly target the family or family behaviors.

To date, the literature offers only 2 examples of diabetes interventions that specifically target and address the Mexican American families’ collective behaviors. The first was a demonstration project carried out in southern Arizona, which used *promotores* to deliver a 12-week program designed to build and reinforce family communication, collective esteem and efficacy, and family support for appropriate food choices and physical activity behaviors. A pre- versus postintervention comparison indicated a significant increase in diabetes knowledge, family efficacy to change food choices, and actual activity behaviors [9]. The other uncontrolled study enrolled 36 Latinx (predominantly Mexican American) families in rural North Carolina in a similar DSME curriculum but the groups were led by a nurse practitioner rather than a *promotore*. Patients showed significant improvement in diabetes knowledge, diabetes self-efficacy, and higher intake levels of healthy foods (ie, fruits and vegetables), and performance of blood glucose testing and foot inspections, while family members demonstrated improved diabetes knowledge and family support compared to baseline [26].

### Group Versus Individual Sessions

The type and setting of these interventions may also be important, and one question that has received some attention is that of group versus individual sessions. One study that tested the “dosing” of an individual DSME found that 16 hours of education with an additional 6 hours of group support was as effective as 52 individual contact hours delivered over 12 months [6]. There are also 4 studies that have directly compared individual with group DSME; three of these led to the conclusion that group education appeared to have a greater impact on diabetes self-management and glycemic control than individual education at 6 and 9 months [27]. However, these were described as poor studies with small numbers and high dropout rates, and there was no significant difference between the interventions at 12 and 18 months. The fourth study did find that patients with T2DM of relatively long duration and HbA1c levels of 7% or higher had improved short-term HbA1c outcomes and a greater likelihood of achieving an HbA1c level below 7% if they were educated using the group approach versus the individual method; however, in this study, the total educator contact time was also greater for the group intervention (8 hours) than for the individual approach (3 hours) [28].

Some families may require extra efforts, such as home visits, telephone reminders, and cultural tailoring, to address the specific obstacles to attendance faced by families [9]. However, other families may need a far more intensive approach to encourage and sustain their participation in DSME, including multifamily groups (MFGs). Two lines of research support the value of MFGs for this purpose. First, randomized controlled studies of MFG in adolescents with type 1 diabetes have shown improvements in diabetes self-care, particularly in patients with poorer glycemic control at baseline [29,30]. Second, the MFG approach has been shown to improve self-management skills in a Mexican American population with chronic mental illness [31]. Given that this study was carried out with a hard-to-reach community of Spanish-speaking Mexican American individuals and that psychiatric symptoms (eg, depression, anxiety, and psychosis) have been identified as major impediments to use diabetes self-management practices [32-35], the results suggest that the MFG approach may be effective in this population.

### Primary Objective

The overall objective of this project is to build the science of T2DM self-management for Latinx patients by constructing a family-centered adaptive intervention to help patients and their relatives enhance their diabetes knowledge and beliefs, increase the use of self-regulation behaviors, and foster social facilitation.

### Hypothesis

There are 4 main hypotheses this study will test. First, subjects randomized to adaptive interventions that begin with nurse-led groups will show greater increases in diabetes self-management behaviors than those who begin with *promotore*-led groups. Second, among nonresponders to either of the first-line approaches, subjects randomized to a multifamily, problem-solving group will show greater improvements than those assigned to a single-family approach. Third, key relative’s participation in treatments will lead to (1) increased patients’ and relatives’ diabetes knowledge; (2) increased diabetes self-efficacy; (3) increased collaborative goal setting by families with health care providers; (4) increased family support; and (5) increased T2DM self-management behaviors. Fourth, subjects who are older, female, less educated, and less acculturated have an alcohol use or psychiatric disorder or have a longer history and greater severity of T2DM will (1) be less likely to have family members participate in treatment and (2) use diabetes self-management skills less often.

## Methods

### Study Overview and Design

**Figure 1:**
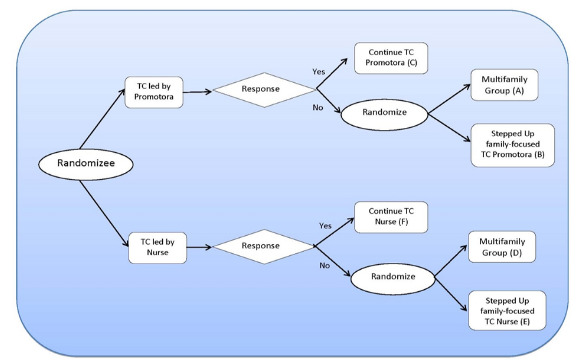
Smart design. TC: Tomando control.

A sequential multiple assignment randomized trial study design evaluates 2 initial interventions for Hispanic adults with T2DM. Figure 1 indicates the study’s procedural flow. First, after baseline assessment, subjects were randomly assigned to receive the *Tomando control* (TC) intervention led either by a community health worker or *promotore* (TC-*promotore*) or by a nurse (TC-nurse). Both of these consist of 6 biweekly 2.5-hour group sessions. After the first 3 intervention sessions (phase 1), preliminary response was measured. Early responders to each intervention continued their same assigned intervention for 3 more sessions. For nonresponders (defined below), participants were rerandomized (phase 2) to receive either an augmented, stepped-up intervention that makes additional efforts to engage family members in the DSME enterprise for 3 more sessions or an MFG intervention designed to individually tailor treatment to address the obstacles encountered in promoting diabetes self-management behaviors. The MFG intervention consisted of 6 additional group sessions.

### Clinical Setting and Patient Population

The study was being conducted in the community served by Olive View-UCLA Medical Center, which is a teaching hospital affiliated with UCLA and part of the Los Angeles County Department of Health Services health care system. It is a safety net facility providing inpatient and outpatient services to the 4 million residents of the San Gabriel, San Fernando, and Antelope Valley regions of Los Angeles County. All patients at Olive View-UCLA Medical Center are under the care of a medical home, which includes full- and part-time staff composed of primary care physicians, residents, interns and medical students, nurses, and nursing assistants. The majority of patients are underinsured or uninsured. Approximately 60% (120,000/200,000) of patients speak Spanish as their primary language, and 60% (120,000/200,000) of patients are of Mexican descent. Among them, over 80% (96,000/200,000) of patients live with their families. The prevalence of T2DM is increased compared to the national average, with 50% (100,000/200,000) of the community affected.

### Subject Eligibility and Recruitment Strategy

Inclusion criteria for study participants include the following: (1) aged at least 18 years, (2) being of Latinx origin speaking fluent Spanish, (3) self-report of type 2 diabetes, suboptimally controlled, defined as having HbA1c values of >7.0%, and (4) living with at least 1 adult family member who is willing to participate. Inclusion criteria of family members are as follows: (1) aged 18 years or older, (2) living in the same patient’s residence, and (3) being of Latinx origin. Exclusion criteria include (1) type 1 diabetes, (2) pregnancy, and (3) significant cognitive impairment.

Participants were recruited from the outpatient primary care clinics of Olive View-UCLA Medical Center. Recruitment began by advertising the study using flyers in the primary care clinics. Interested individuals notified staff in the primary care clinic or the study coordinator. The study coordinator discussed the study and provided further information about the project. With the individual’s permission, key relatives—significant others identified by the individual as among the most important people involved daily in his or her life—were contacted. Both the patient and the family member needed to provide informed consent for either to participate. Subject characteristics at baseline are depicted in Table 1.

**Table 1:** Sample characteristics at baseline (n=330).

Variables			Overall		Nurse (n=165)		Promotore (n=165)		*P* value	
Age (years), mean (SD)			55.33 (8.39)		55.65 (8.29)		55.01 (8.50)		.48	
Education (years), mean (SD)			7.66 (4.21)		7.49 (4.03)		7.84 (4.39)		.46	
**Sex, n (%)**										.27
	Male	144 (43.64)		77 (46.67)		67 (40.61)				
	Female	186 (56.36)		88 (53.33)		98 (59.39)				
**Marital status, n (%)**										.38
	Never married	92 (27.88)		50 (30.30)		42 (25.45)				
	Married	164 (49.70)		78 (47.27)		86 (52.12)				
	Divorced	34 (10.30)		14 (8.48)		20 (12.12)				
	Widowed	15 (4.55)		7 (4.24)		8 (4.85)				
	Separated	25 (7.58)		16 (9.70)		9 (5.45)				
**Employment, n (%)**										.46
	Full-time	41 (12.42)		22 (13.33)		19 (11.52)				
	Part time	83 (25.15)		48 (29.09)		35 (21.21)				
	Homemaker	64 (19.39)		29 (17.58)		35 (21.21)				
	Retired	22 (6.67)		12 (7.27)		10 (6.06)				
	Disabled	58 (17.58)		28 (16.97)		30 (18.18)				
	Unemployed	62 (18.79)		26 (15.76)		36 (21.82)				

### Characteristics and Training of DSME Leaders

The *promotores* were recruited from the workforce of Olive View-UCLA Medical Center (3 women and 1 man). They were of Hispanic origin and spoke fluent Spanish and English. The 3 RNs were employees at Olive View-UCLA Medical Center. All 3 spoke English and Spanish fluently. Two of the 3 RNs were of Hispanic origin. Both *promotores* and RNs were trained and certified in the Stanford Chronic Disease Self-Management program. This training included a 4-day onsite workshop in Palo Alto, California, followed by a cross-training webinar on diabetes self-management education and 2 sets of practice groups conducted at Olive View-UCLA Medical Center designed to ensure fidelity to the treatment model.

### Initial Intervention: Tomando Control

The initial intervention, called “Taking Control of your Diabetes” or in Spanish “*Tomando Control de su diabetes*,” consisted of 6 biweekly sessions lasting 2.5 hours each led either by 2 trained *promotores* (*promotore*-led group) or by RNs (RN-led group). Each session included 10-12 patients. Patients’ families or friends were encouraged to attend sessions, which were held at Olive View-UCLA Medical Center. Each participant received a copy of the companion book, an audio relaxation tape, and an audio exercise tape with a booklet to supplement the material covered during the workshop sessions. The following topics were covered in sessions: (1) techniques to deal with the symptoms of diabetes and associated condition, (2) appropriate exercise, (3) healthy eating, (4) correct use of diabetes medications, and (5) working more effectively with health care providers in a collaborative partnership. Patients were expected to make weekly action plans, share experiences, and help each other solve problems they encountered in creating and carrying out their self-management strategies. The classes were highly participative and allowed participants to show mutual support and develop confidence in their abilities to manage their health. The program is based on the Stanford School of Medicine’s Chronic Disease Management Program developed by Lorig et al [36] and culturally adapted by Mauldon et al [37]. Similar program content was delivered in each group, with the only difference being the type of trainer: *promotore* versus RN.

Early response status was determined after the third TC session (ie, 6 weeks after starting the intervention). An independent evaluator blind to study condition used the Summary of Diabetes Self-Care Activities (SDSCA) to assess diabetes self-management behavior. A subject was considered an early responder if he/she showed a gain of 50% over baseline on the SDSCA. Early responders continued to receive the TC intervention that they were originally assigned. Nonresponders, those subjects who did not show a gain of 50% over baseline, were rerandomized to receive either the stepped-up, family-focused TC approach or the MFG. The following sections describe these 2 interventions.

### Second Intervention: Family-Focused TC Approach

For those subjects who were randomly assigned to receive the augmented TC, the TC clinician (either a *promotore* or nurse, depending on the original group assignment) conducted up to 3 home visits that were designed to explain the purpose of the TC intervention to a key relative so as to reengage the family member in the group process. Each visit was carefully scripted to address the family’s role in helping the family member with diabetes. The first home visit was focused on the family’s reaction to having a loved one with diabetes and was designed to elicit how the family functioning had changed since the diagnosis was made. The second session aimed to help the family set realistic goals for participation in TC including the length of the groups and the expectations of the family in using the material covered in the group to effect behavior change. Logistical details (eg, which family member would accompany the patient to the group, how they would get to the group, and so on) were identified and obstacles to attendance were resolved. The third session solidified the gains made in the first 2 sessions as well as emphasized to the family that the *promotore* or nurse was available for crises or problem resolution if last-minute difficulties arose prior to the TC group. These engagement strategies have been very effective in increasing the participation of family members in group therapy [38].

### Second Intervention: Multifamily Problem-Solving Group Approach

#### Overview

The other treatment assignment for nonresponders to TC was MFG. As described by McFarlane [39], standard MFG consists of 3 components: three initial “joining” sessions conducted with each of the families separately, a 1-day (6 hours) educational workshop, and ongoing MFG sessions. In the MFG used in this project, the joining sessions and educational workshop were performed as described in the MFG manual. However, the ongoing multifamily sessions in this project differed from standard MFG in which it was explicitly organized around the obstacles to diabetes self-management identified in family interviews. Each MFG session included structured activities that ensured participation by all families in each content area, gave information, carried out problem-solving exercises, and provided skills training focused on increasing individuals’ diabetes self-management behaviors.

#### Joining Sessions

The 3 joining sessions offered each family an individualized approach designed to facilitate the process of engaging in treatment. The sessions were conducted by a bilingual, bicultural *promotore* or nurse, depending on the subject’s initial group assignment. The sessions were designed to familiarize all members of the family with the therapists who conducted the ongoing MFG sessions and to educate them about the need for ongoing treatment. The sessions also helped the family identify and overcome the obstacles to successful diabetes self-management. All family members were given the opportunity to describe their understanding of their ill relative’s problem and were also given factual information based on current knowledge about diabetes.

#### Educational Workshop

The educational workshop is a 6-hour program that provides information about the etiology, biology, genetics, symptoms, and treatment of T2DM. Much of this material will be covered in the TC groups; however, the educational workshop was made available to those MFG family members who did not attend the TC sessions. It was conducted by the MFG clinician along with one of the authors (AK). They used the Spanish translation of the standardized videotape and coping skills guidelines developed by the author. The material is presented in basic, elementary school level Spanish with little use of idioms or colloquialisms. Presentations were made both visually and orally, including the use of role plays to allow family members with poor reading skills to adequately assimilate the material.

#### Ongoing MFG Sessions

Following the joining sessions and educational workshop, each cohort of 5-6 families met twice monthly for 3 months (6 sessions total). All sessions were co-led by the clinicians who conducted the 3 joining sessions. The first MFG session was focused on teaching problem-solving skills. Participants learned the mechanics of the problem-solving method and how to apply it to various problem situations. The method involves 7 steps as follows: define the problem, set goals, generate alternatives, evaluate each, select one, implement it, and evaluate outcomes. The subsequent 5 sessions were structured in the usual MFG fashion. This included a brief sharing period followed by group discussion. The discussion consisted of the sharing of personal experiences, identification of a problem situation, and the use of the problem-solving method to address the situation within the context of general group support. The group ended with review of the gains made by group members that week.

### Data Collection and Measures

Table 2 lists the measures to be used at each assessment point. Assessments were done at baseline and at 3, 6, and 12 months. In addition, the primary outcome variable (ie, diabetes self-management behaviors) was measured 6 weeks after the start of the first phase of the study to determine progress over time and the need for a change in treatment strategy. All measures were collected by a research assistant blind to the experimental conditions. Characteristics of the sample were measured at the initial visit including various demographic and clinical information.

The primary outcome is the difference in response scores of the SDSCA between the 4 groups at 3, 6, and 12 months after baseline [40]. The SDSCA is a well-validated instrument with excellent psychometric properties in several languages including Spanish [41,42]. It is the instrument most frequently used for assessing the acquisition of diabetes self-management behaviors [43]. The SDSCA is formatted to ask on how many of the previous 7 days the individual performed recommended self-management activities: eating healthy foods, following a diabetic diet, exercising, self-monitoring of glucose, and caring for one’s feet. A weekly self-care score ranging from 0 to 7 is generated, with higher scores corresponding to greater number of days carrying out the recommended behaviors.

Several secondary outcomes were also measured in this study. Diabetes self-efficacy, defined as the confidence of a person with diabetes to manage diet, exercise, track blood glucose levels, and overall control their diabetes, was measured using the 8-item Stanford Self-Efficacy Scale. Scores range from 1 (no confidence) to 10 (total confidence). The internal reliability of the Spanish language version of this measure is 0.85 [44]. Diabetes knowledge was measured using the Spoken Knowledge in Low Literacy Patients with Diabetes scale, a 10-item scale that assesses knowledge of glucose management, lifestyle modifications, recognition, and treatment of hyper- and hypoglycemia, and activities to prevent long-term consequences of the disease. Higher scores indicate better knowledge about diabetes, and the measure has been found to have excellent validity and reliability [45]. The patient’s degree of family support was measured using the 17-item Diabetes Family Support Behavior Checklist, which uses a 5-point Likert scale to assess the patients’ perceptions about their relatives’ support in medication taking, glucose monitoring, exercise, and diet [46]. Cronbach *α*s have been reported between 0.64 and 0.71 in Hispanic subjects with diabetes [26,47]. Collaborative goal setting was measured with the Spanish version of the Patient Assessment of Chronic Illness Care [48], a 20-item survey for evaluating the quality and patient-centeredness of chronic illness care received by the patient according to the Chronic Care Model paradigm [49]. The Spanish language translation of this instrument has demonstrated high reliability, internal consistency, and test-retest reliability [50]. Treatment session attendance was assessed for both patients and their relatives, and the percentage of treatment sessions attended by the key relative was used to analyze relationships with other outcomes. Glycemic control, defined as HgA1c level, was tested using a Bayer A1C NOW kit (Bayer Healthcare) using finger stick blood taken by a phlebotomist. Levels greater than 7.0% are considered poor glycemic control, and levels greater than 10.0% are considered uncontrolled diabetes [51]. Height, weight, BMI, and blood pressure were measured using standard procedures.

**Table 2:** Study measures.

Variables	Measure	Content	Schedule^a^
Demographics	UCLA Client Data Inventory Cuellar acculturation scale Structured Clinical Interview for DSM-IV (SCID-I)	Age, gender, marital status, work status, educational level, acculturation level, and medical/psychiatric disorders	T1
Diabetes self-management	Revised Summary of Diabetes Self-Care Activities (SDSCA)	Assesses adherence to diet, exercise, self-monitoring, medications, and foot care	T1-T4 and week 6
Diabetes self-efficacy	Stanford Self-Efficacy Scale	Measures confidence to self-manage diabetes	T1-T4
Diabetes knowledge	Spoken Knowledge in Low Literacy Diabetes Patients (SKILLD)	Assesses knowledge of T2DM and its management	T1-T4
Family support	Diabetes Family Support Behavior Checklist	Assesses family support for T2DM self-management	T1-T4
Collaborative goal setting	Patient Assessment of Chronic Illness Care (PACIC)	Evaluates quality and patient centeredness of received treatment	T1-T4
Treatment attendance	Treatment Attendance Log	The percentage of sessions attended by patient/relative	T3
T2DM control	Bayer A1C NOW kit	Hemoglobin A1c level	T1-T4

^a^Data collection schedule: T1: baseline; T2: 3 months; T3: 6 months; T4: 12 months.

### Statistical Analysis

Data analysis will be conducted by intent-to-treat with participants analyzed according to their randomized intervention regardless of attendance. Our primary analytical tool will be the generalized linear mixed model (GLMM) [52,53]. GLMMs accommodate multiple outcome types in a unified framework via an appropriate link function (identity link for continuous normally distributed measures; logistic link for binary measures); allow for both fixed and time-varying covariates; automatically handle missing data, producing unbiased parameter estimates provided observations are missing at random; and appropriately account for the correlations induced by repeated measurements within subjects. We will fit GLMMs with fixed effects for the intercept, time (baseline, 3, 6, and 12 months), group, and a group-by-time interaction term, where the definition of group depends on the hypothesis and an unstructured covariance matrix. Model diagnostics will be used to determine suitability of more parsimonious (eg, autoregressive) correlation structures and nonlinear (eg, quadratic) effects for time. A sample size of 330 patients will provide 90% power to detect a difference of 0.29 in the score of SDSCA between the study groups. Subgroup analysis will be performed to determine if some moderators differentially affect the outcome such as patients’ age, gender, educational levels, duration of diabetes, and baseline HbA1c levels.

### Ethics Approval

All participants signed an informed consent form. The trial protocol was reviewed and approved by the Institutional Review Board of the David Geffen School of Medicine at UCLA (#16-000434) and the Education and Research Institute of Olive View-UCLA Medical Center (#882988). Initial approvals were received in November 2016, and yearly renewal approvals were received throughout the course of the study. The 1-year follow-up assessments were completed in late 2021.

## Results

This study was funded by the National Institute of Nursing Research in June 2016 for a period of 5 years. Institutional review board approval was obtained in November 2016. Between March 2017 and September 2020, a total of 330 patients were recruited from the outpatient primary care clinics of Olive View-UCLA Medical Center, with a brief hiatus between May 2020 and July 2020 due to COVID-19 restrictions. The study interventions were completed in December 2020. Data collection began in March 2017 and was completed in December 2021. Data analysis is expected to be completed in Spring 2023, and results will be published in Fall 2023.

## Discussion

### Principal Findings

This paper describes the protocol of a sequential multiple assignment randomized trial to better understand how various factors in the design and implementation of a DSME program can affect the outcomes of participants with T2DM.

The first question we address is whether RNs are more effective than *promotores* in leading DSME programs for Latinx patients with T2DM. This study represents a direct test of the hypothesis that Latinx subjects assigned to RN-led groups will perform more diabetes self-management behaviors than subjects assigned to *promotore*-led groups. The findings of this aspect of the study could have important economic implications, particularly if subjects in RN-led groups do not achieve better outcomes than subjects in *promotore*-led groups because of the greater cost and lower availability of RNs than those of *promotores*.

The design of this study will also further our understanding of the efficacy of interventions that use MFGs and how these are compared with interventions that focus on individual families. The reasons that patients may fail initial DSME interventions are numerous and varied, such that it may be difficult for any individual educator—professionally trained or otherwise—to be able to address all barriers that arise for a patient. The incorporation of multiple families with similar backgrounds in the same community provides the opportunity for participants to offer their combined, cumulative perspectives and experiences in order to problem solve and support their peers. This is important not only because of the increased likelihood of identifying reasonable solutions but also because it could help build a stronger sense of self-efficacy and agency among participants while also decreasing stigma, shame, and isolation. Furthermore, because sessions are intended to address behaviors and beliefs among the entire family, we hope to gain additional insight into how these dynamics influence outcomes for participants in this program.

This study will also measure various demographic variables including age, gender, marital status, working status, educational level, acculturation level, comorbid medical and psychiatric disorders, and severity of diabetes. These will also be analyzed to explore whether particular characteristics of the sample can serve as potential predictors of outcomes.

### Comparison to Prior Work

There have been some studies that compared outcomes of patients with T2DM when counseled by RNs versus community health workers (the equivalent of *promotores*). The randomized trial by Babamoto et al directly compared the effectiveness of community health workers with case management and standard provider care in Latinx patients with T2DM for 6 months. In that study, patients in the community health worker group performed better than the other groups in several outcomes, namely higher retention rates, greater reduction in HbA1c concentrations, and medication adherence [16]. Kim et al compared outcomes in Korean Americans with T2DM, finding that patients counseled by community health workers had greater decreases in HbA1c concentrations than RN-counseled patients [54].

Several other studies using different methodological approaches have shown that interventions led by community health workers were cost effective and result in substantial reductions in diabetes complications. Ryabov et al estimated that interventions led by community health workers could result in an absolute reduction in projected probability of lifetime occurrence of nephropathy by 5.9%, neuropathy by 3.4%, retinopathy by 2.6%, and coronary artery disease by 3.8% [55]. Moreover, Brown et al suggested that *promotores* were most cost-effective among Latinx patients aged 50-65 years [56]. The benefits of community health workers extend to other ethnicities. For example, Huang et al found that home visits conducted by community health workers were highly cost-effective in a randomized study of 268 patients with T2DM in American Samoa [57]. We expect that our study will be particularly useful in building on this knowledge because the educational programming is standardized between both groups, with all participants receiving the same evidence-based intervention.

### Strengths and Limitations

Strengths of this study include its randomized design and inclusion of enough patients to allow for adequate statistical power and subgroup analysis. This study uses the SDSCA, which is a well-validated instrument to assess diabetes self-management behavior. It is the most widely used self-report instrument for measuring diabetes self-management in US adults [41]. In an appraisal of 26 psychometric tools of diabetes education, Eigenmann et al identified the SDSCA as 1 of 3 tools that met all expected criteria for validity, reliability, relevance, feasibility and acceptability, and responsiveness to change [58].

One potential limitation of this study is that the investigators were aware of the patients’ group assignment, and therefore bias in favor of any particular group cannot be excluded. However, every effort was made to ensure blinding. For instance, *promotores* and RNs were not aware of the purpose of the trial. An independent evaluator blind to study conditions was responsible for administering all study measures. A second limitation is the short duration of the trial (ie, 6 sessions over 3 months). However, many years of clinical experience and research data suggest that 6 intervention sessions are sufficient to result in clinically significant changes in diabetes self-management behavior [59].

### Conclusions and Future Directions

The findings of this trial should contribute to our understanding of the roles of community health workers, RNs, and family members in this area and may have important economic implications. Future research will focus on understanding the factors that affect self-management for Latinx patients with T2DM and will provide valuable information toward constructing an adaptive intervention that will help to determine which treatment strategies work to improve diabetes self-management behaviors most efficiently and for whom. Given the ever-increasing prevalence of T2DM, achieving better control of diabetes and lowering the associated medical complications experienced disproportionally by Latinx patients is a public health priority for a group that has long been underserved by current research and practice.

## Appendix

Multimedia Appendix 1 Peer-review reports from the Nursing and Related Clinical Sciences (NRCS) Study Section - Healthcare Delivery and Methodologies Integrated Review Group - Center for Scientific Review (National Institutes of Health, USA).

## Abbreviations

DSME: diabetes self-management education
GLMM: generalized linear mixed model
HbA1c: hemoglobin A1c
MFG: multifamily group
RN: registered nurse
SDSCA: Summary of Diabetes Self-Care Activities
T2DM: type 2 diabetes mellitus
TC: Tomando control

## Data Availability

Deidentified data will be made available to all appropriate requests from interested researchers at the time of results publication.
