# Supplementary material for: Improving Self-management of Type 2 Diabetes in Latinx Patients: Protocol for a Sequential Multiple Assignment Randomized Trial Involving Community Health Workers, Registered Nurses, and Family Members
Source: JMIR Res Protoc. 2023 Jan 16;12:e44793. doi: 10.2196/44793 (PMC9887518; doi:10.2196/44793)
Supplement: Multimedia Appendix 1 [file resprot_v12i1e44793_app1.pdf]

SUMMARY STATEMENT
(Privileged Communication)

PROGRAM CONTACT:
Karen Huss
301.594.5970
hussk@mail.nih.gov

Release Date: 10/28/2015

Application Number: 1 R01 NR015809-01A1

Principal Investigator

KOPELOWICZ, ALEX J MD, DPH

Applicant Organization: UNIVERSITY OF CALIFORNIA LOS ANGELES

Review Group: NRCS
Nursing and Related Clinical Sciences Study Section

Meeting Date: 10/08/2015
Council: JAN 2016
Requested Start: 04/01/2016
RFA/PA: PA14-112
PCC: ACCKH

Project Title: Using Multifamily Groups to Improve Family-Centered Self-Management of Type 2 Diabetes among Mexican Americans
SRG Action: Impact Score: 20 Percentile: 5
Next Steps: Visit http://grants.nih.gov/grants/next_steps.htm
Human Subjects: 30-Human subjects involved - Certified, no SRG concerns
Animal Subjects: 10-No live vertebrate animals involved for competing appl.
Gender: 1A-Both genders, scientifically acceptable
Minority: 2A-Only minorities, scientifically acceptable
Children: 1A-Both Children and Adults, scientifically acceptable
Clinical Research - not NIH-defined Phase III Trial

Project Year	Direct Costs Requested	Estimated Total Cost
1	345,075	434,795
2	346,418	436,487
3	349,611	440,510
4	347,354	437,666
5	315,669	397,743
TOTAL	1,704,127	2,147,201

ADMINISTRATIVE BUDGET NOTE: The budget shown is the requested budget and has not been adjusted to reflect any recommendations made by reviewers. If an award is planned, the costs will be calculated by Institute grants management staff based on the recommendations outlined below in the COMMITTEE BUDGET RECOMMENDATIONS section.

1R01NR015809-01A1 KOPELOWICZ, ALEX

RESUME AND SUMMARY OF DISCUSSION: This application proposes a Sequential Multiple Assignment Randomized Trial design to evaluate culturally adapted practices to improve self-management behaviors and self-efficacy among Mexican-American patients with Type 2 diabetes (T2DM). The project retains the strong features of the previous submission, including the use of a family-focused approach to improve program attendance and outcomes and an innovative SMART design that will allow for testing multiple interventions across time. The resubmission is also strengthened by changes that are responsive to most of the concerns identified in the previous review. Detracting only slightly from these strong points is a remaining concern regarding the inclusion of a broad patient age range of participants. Overall, the proposed research is considered very important and likely to have a high impact on the field of diabetes health disparities.

DESCRIPTION (provided by applicant): This R-01 grant proposal is a response to PA-14-112 "A Family-Centered Self-Management of Chronic Conditions." The project (a) evaluates four culturally adapted, family-based treatments designed to improve health status, self-management behaviors and self-efficacy among Mexican-Americans with Type 2 diabetes using a Sequential, Multiple Assignment Randomized Trial in a public health setting, and (b) tests hypotheses about the applicability of Ryan and Sawin's Individual and Family Self-Management Theory, the conceptual foundation for the intervention's key constructs, to the study of diabetes self-management in this population. This conceptual model was selected for study because its emphasis on the integration of family members as partners in care while promoting illness self-management is highly germane to the study population and to the proposed intervention. Spanish speaking, Mexican-American patients with Type 2 diabetes and their families will be recruited from a primary care clinic affiliated with a large, urban public hospital located in a predominantly Mexican American area of Los Angeles. In the first phase of the study, subjects will be randomly assigned to six 2.5 hour sessions (three months) of either: 1) Tomando Control de su Diabetes (TC), a culturally tailored, community-based, Diabetes Self-Management program delivered in a group format by community health workers (promotoras) working with individual patients and families; or 2) TC delivered by health professionals (licensed nurses). Evaluations will be made at baseline, three months, six months and 12 months. After six weeks of treatment (at the midway point of the intervention), subjects will be assessed for improvement in diabetes self-management behaviors (the primary outcome). In the second phase of the study, those subjects who have improved their diabetes self-management behaviors by 50% over baseline will be continued in their assigned treatment for the duration of three months. Those subjects who do not meet this target will be re-randomized to receive either: 1) an augmented version of TC that has a specific focus on engaging family members; or 2) a multifamily group treatment led by nurses specially trained in teaching diabetes self-management skills for an additional three months. Subjects will be assessed on setting and adhering to self-management goals related to diabetes; self-management behaviors including adhering to the ADA diet, engaging in exercise activities, monitoring glucose levels and taking prescribed hypoglycemic medications; diabetes self-efficacy; and hemoglobin A1c levels. Family members will be assessed during these same time frames to determine their knowledge of diabetes care; levels of collaborative goal setting with their ill relative and the health care provider; and levels of family support. The results will advance the understanding of the factors that affect self-management for Mexican-Americans with Type 2 diabetes, and will provide valuable information towards constructing an adaptive intervention that will help to determine which treatment strategies work to improve diabetes self-management behaviors most efficiently and for whom.

PUBLIC HEALTH RELEVANCE: The overall objective of this application is to construct an adaptive intervention that integrates family members and patients as partners in care while promoting diabetes self-management for Mexican Americans with Type 2 diabetes. The project incorporates four evidence-based, culturally tailored treatments using a Sequential, Multiple Assignment Randomized Trial to help

determine what sequence of intervention strategies work most efficiently and for whom. Given the ever increasing prevalence of Type 2 diabetes, achieving better control of diabetes and lowering the associated medical complications experienced disproportionally by Mexican Americans is a public health priority.

CRITIQUE 1

Significance: 1
Investigator(s): 1
Innovation: 1
Approach: 3
Environment: 1

Overall Impact: This study aims to advance the science of family centered self-management for Mexican-American patients with Type 2 diabetes by using a Sequential Multiple Assignment Randomized Trial design to evaluate culturally adapted practices for promoting diabetes self-management. This is an outstanding proposed study, one that is highly likely to make a sustained and powerful impact. Major strengths include the significance of the problem, the responsiveness of the proposed study to the program announcement, the innovative study design and the well-reasoned selection of interventions to study. The investigators are well-qualified and present a thoughtful and convincing work plan for achieving the goals of the study. In this resubmission, the investigators were responsive to previous critiques. The sole weakness in the approach noted by this reviewer is the inclusion of a wide age range, which may result in attenuated intervention effects. Offsetting this concern, however, are the advantages in generalizability that come from such a broad inclusion range.

1. Significance:

Strengths

- Increasing prevalence of Type 2 diabetes in Mexican-Americans and large, disproportionate burden arising from suboptimal control of diabetes in this population heightens the significance.
- Clear and convincing rationales for interventions selected for comparison.
- Will provide test of Individual and Family Self-Management Theory.

Weaknesses

- None noted.

2. Investigator(s):

Strengths

- PI is Chief of Psychiatry at Olive View UCLA Medical Center (OVMC) and an established investigator with ample relevant experience and expertise with psychosocial intervention studies of Mexican-Americans, including studies of the Multifamily Group (MFG) approach.
- Dr. Ruiz is an expert on the impact of culture in the treatment of chronic disease. She is particularly well-qualified for training and monitoring the promotoras and nurses providing the interventions.
- Drs. Namby and Wali provide complementary expertise in statistics and internal medicine, respectively.

- Investigative team includes highly experienced research coordinator.

Weaknesses

- None noted.

3. Innovation:

Strengths

- Application of Sequential, Multiple Assignment Randomized Trial (SMART) design to family-centered self-management studies.
- Relatively few diabetes self-management education (DSME) interventions have targeted Hispanic families along with patients.
- DSME intervention that targets family's collective behaviors.
- Will extend study of multifamily group (MFG) approach to families of patients with type-2 diabetes.

Weaknesses

- None noted.

4. Approach:

Strengths

- Study design permits assessing multiple attributes of interventions--including format (group vs. individual), type of educator and treatment target.
- Plausible and theory-based rationale for identifying effect moderators under consideration (in Aim 4).
- Sound measurement and randomization plans.
- Attention to monitoring and maintaining intervention fidelity.
- Well-developed statistical analysis plans.

Weaknesses

- The inclusion of such a broad patient age range (18-65 years old) should be better justified by the investigators (a concern also raised by a previous reviewer), given the pros and cons of such a decision. A broad range can attenuate the apparent effect of the intervention, particularly if the effectiveness of family-centered self-management interventions varies with the developmental life stage of the patient. On the other hand, age is nominated as an effect moderator in Aims 3 and 4, so permitting a wide range of ages facilitates assessing age as an effect moderator.

5. Environment:

Strengths

- OVMC is well-suited to achieve recruitment goals and to otherwise support proposed research.
- UCLA SIStat Data Core will provide data entry, management and analysis support.

Weaknesses

- None noted.

Protections for Human Subjects:

Acceptable Risks and/or Adequate Protections

- No concerns were identified.

Data and Safety Monitoring Plan (Applicable for Clinical Trials Only):

Acceptable

- No concerns were identified.

Inclusion of Women, Minorities and Children:

- Sex/Gender: Distribution justified scientifically
- Race/Ethnicity: Distribution justified scientifically
- Inclusion/Exclusion of Children under 21: Including ages < 21 justified scientifically
- No concerns were identified.

Vertebrate Animals:

Not Applicable (No Vertebrate Animals)

Biohazards:

Not Applicable (No Biohazards)

Resubmission:

- Mostly responsive to previous critiques.

Resource Sharing Plans:

Not Applicable (No Relevant Resources)

Budget and Period of Support:

Recommend as Requested

- No concerns were identified.

CRITIQUE 2

Significance: 3
Investigator(s): 1
Innovation: 2
Approach: 2
Environment: 1

Overall Impact: This is a resubmission of a proposal that aims to test four family-based treatments to improve self-management behaviors and self-efficacy among Mexican Americans with type 2 diabetes. Type 2 diabetes is an important public health concern, and Latinos are particularly impacted. This is a

strong application. The application proposes an innovative SMART design that will allow for testing multiple interventions across time, and will use a stepped-up family engagement strategy to improve program attendance and outcomes. Another key strength is that the intervention will test family-based approaches, which have been shown to be particularly successful in Latino populations. The use of the Family Self-Management Theory appears appropriate. The intervention represents an important advancement in the field.

1. Significance:

Strengths

- Type 2 diabetes is a growing public health crisis and Latinos have higher incidence and morbidity than non-Latino whites.
- Little is known about the success of family-based interventions for Latinos.
- If successful, the study could lead to a greater understanding of the effectiveness of family-based interventions addressing type 2 diabetes.
- Previous evaluations have tested family-based approaches for addressing mental illness in Mexican Americans and type 1 diabetes in adolescents, but key knowledge about whether the approach is effective in improving self-management practices among Latinos with uncontrolled diabetes is lacking.
- The intervention addresses the key issue with success of any self-management program, that is, adherence with the program components.

Weaknesses

- Given the comparison between a nurse-led and promotora-led program, it would appear that a ‘missed opportunity’ may be the inclusion of a cost-effectiveness assessment. Moreover, the nurse may have been integrated with the electronic health record and more access to the medical care team. While the promotora is considered lower cost, from a human resource perspective, a lack of understanding of the cost-effectiveness may hinder the advancement of this field.

2. Investigator(s):

Strengths

- The investigator team appears strong, with expertise in nursing, and a history of leading promotora programs. Notably, a co-I for the project, Dr. Wali, is also the chief of medicine at Olive View-UCLA Medical Center, whose primary care clinics will serve as recruitment sites for the study.
- The PI has conducted other multi-family group interventions involving Mexican American populations (addressing mental illness).

Weaknesses

- A minor weakness is that the expertise of the PI is mental health; the inclusion of an investigator with expertise in diabetes prevention and assessment may be useful.

3. Innovation:

Strengths

- The study will use an innovative, SMART, design, which will allow for the testing of multiple interventions across time.

Weaknesses

- None.

4. Approach:

Strengths

- The approach is well-founded in research. Latinos highly value family, thus testing family-based intervention is appropriate.
- The use of the Family Self-Management Theory appears appropriate given the emphasis on family in the intervention.

Weaknesses

- One minor concern is a 10% attrition used in estimates for power calculations. Attrition could be higher given the intensity of the intervention program. Moreover, there could very well be differential attrition by group – it is unclear whether and how the team plans to assess or monitor this.
- A second minor point is that the recruitment goal of 330 may be too small to conduct meaningful analysis of moderators of the intervention.

5. Environment:

Strengths

- UCLA is the primary applicant and has substantial research resources.
- Olive View-UCLA treats about 10,000 individuals with type 2 diabetes each year, and about 60% of their patients are Mexican American. The chief of medicine is a co-I on the study. It appears to be in ideal environment for recruiting eligible patients.
- The participation of the two *promotoras* will be facilitated by the Latino Behavioral Health Institute, which has a long and successful history of training and mentoring *promotoras* who serve in community settings throughout the Mexican American neighborhood of Los Angeles County. The promotoras will already be trained in Tomando Control de Su Salud.

Weaknesses

- It is unclear how geographically dispersed the clinics and patients are – and what challenges this might pose to recruitment and retention.

Protections for Human Subjects:

Acceptable Risks and/or Adequate Protections

- No concerns were identified.

Data and Safety Monitoring Plan (Applicable for Clinical Trials Only):

Acceptable

- No concerns were identified.

Inclusion of Women, Minorities and Children:

- Sex/Gender: Distribution justified scientifically
- Race/Ethnicity: Distribution justified scientifically

- Inclusion/Exclusion of Children under 21: Including ages < 21 justified scientifically
- No concerns were identified.

Vertebrate Animals:

Not Applicable (No Vertebrate Animals)

Biohazards:

Not Applicable (No Biohazards)

Resubmission:

- No concerns were identified.

Resource Sharing Plans:

Not Applicable (No Relevant Resources)

Budget and Period of Support:

Recommend as Requested

- No concerns were identified.

CRITIQUE 3

Significance: 1
Investigator(s): 1
Innovation: 1
Approach: 2
Environment: 1

Overall Impact: This is an exciting proposed study to work with Latinos, primarily of Mexican origin, who have Type 2 diabetes and expands the intervention to non-adherent participants to include their families as motivators. Drs. Kopelowicz and Ruiz have strong established track records in working with this proposed population. It is anticipated that this RCT design will significantly inform familial interventions for this population, making it applicable beyond diabetes, which is salient in reducing health disparities burdens.

1. Significance:

Strengths

- The PI presents clear rationale for the need for a family-focused diabetes self-management intervention.
- Testing a familial model to address diabetes management adherence holds promise as a disease preventative intervention with the family as well, which is significant in this population that bears a disproportionate burden.

Weaknesses

- No score driving weaknesses.

2. Investigator(s):

Strengths

- This is a strong team that has worked with the Latino population in other studies. They have made meaningful scientific contributions in the various areas, diabetes, family, methods etc., thus proving confidence that they will be able to conduct the research and disseminate it in a timely manner.

Weaknesses

- No score driving weaknesses.

3. Innovation:

Strengths

- This is a highly innovative application in that it is using multiple evidenced-based interventions coupled with a sequential multiple assignment randomization, that should strengthen the interpretation of rich data related to a best practice familial intervention.

Weaknesses

- No score driving weaknesses.

4. Approach:

Strengths

- This is an exciting application. It is anticipated that this RCT design will significantly inform familial interventions for this population, making it applicable beyond diabetes, which is salient in reducing health disparities burdens.

Weaknesses

- None noted.

5. Environment:

Strengths

- This is an outstanding research environment to conduct this study that includes a letter of support from the Latino Behavioral Health Institute.

Weaknesses

- No score driving weaknesses.

Protections for Human Subjects:

Acceptable Risks and/or Adequate Protections

- No concerns were identified.

Data and Safety Monitoring Plan (Applicable for Clinical Trials Only):

Acceptable

  - No concerns were identified.

Inclusion of Women, Minorities and Children:

- Sex/Gender: Distribution justified scientifically
- Race/Ethnicity: Distribution justified scientifically
- Inclusion/Exclusion of Children under 21: Including ages < 21 justified scientifically
- No concerns were identified.

Vertebrate Animals:
Not Applicable (No Vertebrate Animals)

Biohazards:
Not Applicable (No Biohazards)

Resubmission:

- No concerns were identified.

Resource Sharing Plans:
Not Applicable (No Relevant Resources)

Budget and Period of Support:
Recommend as Requested

- No concerns were identified.

THE FOLLOWING SECTIONS WERE PREPARED BY THE SCIENTIFIC REVIEW OFFICER TO SUMMARIZE THE OUTCOME OF DISCUSSIONS OF THE REVIEW COMMITTEE, OR REVIEWERS' WRITTEN CRITIQUES, ON THE FOLLOWING ISSUES:

PROTECTION OF HUMAN SUBJECTS (Resume): ACCEPTABLE

INCLUSION OF WOMEN PLAN (Resume): ACCEPTABLE

INCLUSION OF MINORITIES PLAN (Resume): ACCEPTABLE

INCLUSION OF CHILDREN PLAN (Resume): ACCEPTABLE

COMMITTEE BUDGET RECOMMENDATIONS: The budget was recommended as requested.

NIH has modified its policy regarding the receipt of resubmissions (amended applications).
See Guide Notice NOT-OD-14-074 at http://grants.nih.gov/grants/guide/notice-files/NOT-OD-

14-074.html. The impact/priority score is calculated after discussion of an application by averaging the overall scores (1-9) given by all voting reviewers on the committee and multiplying by 10. The criterion scores are submitted prior to the meeting by the individual reviewers assigned to an application, and are not discussed specifically at the review meeting or calculated into the overall impact score. Some applications also receive a percentile ranking. For details on the review process, see http://grants.nih.gov/grants/peer_review_process.htm#scoring.

MEETING ROSTER

Nursing and Related Clinical Sciences Study Section
Healthcare Delivery and Methodologies Integrated Review Group
CENTER FOR SCIENTIFIC REVIEW
NRCS
October 08, 2015 - October 09, 2015

CHAIRPERSON

HEITKEMPER, MARGARET MCLEAN, RN, PHD, FAAN
PROFESSOR AND CHAIR
DEPARTMENT OF BIOBEHAVIORAL NURSING
AND HEALTH SYSTEMS
SCHOOL OF NURSING
UNIVERSITY OF WASHINGTON
SEATTLE, WA 98195

MEMBERS

AOUIZERAT, BRADLEY E, PHD, MS
PROFESSOR AND DEPUTY DIRECTOR
BLUESTONE CENTER FOR CLINICAL RESEARCH
DEPARTMENT OF ORAL AND MAXILLOFACIAL SURGERY
NEW YORK UNIVERSITY
NEW YORK, NY 10010

BAKITAS, MARIE ANNE, DNSC, FAAN
PROFESSOR
MARIE O'KOREN ENDOWED CHAIR
CENTER FOR PALLIATIVE AND SUPPORTIVE CARE
UNIVERSITY OF ALABAMA AT BIRMINGHAM
BIRMINGHAM, AL 35294

BURGENER, SANDRA C, RN, PHD, FAAN *
ASSOCIATE PROFESSOR EMERITA
DEPARTMENT OF BIOBEHAVIORAL HEALTH NURSING
UNIVERSITY OF ILLINOIS
URBANA, IL 46202

CLARKE, SEAN P, PHD
PROFESSOR AND ASSOCIATE DEAN
CONNELL SCHOOL OF NURSING
BOSTON COLLEGE
CHESTNUT HILL, MA 02467

CORONADO, GLORIA D, PHD *
MITCH GREENLICK ENDORSED SENIOR INVESTIGATOR
KAISER FOUNDATION
RESEARCH INSTITUTE
PORTLAND, OR 97227

CORWIN, ELIZABETH JEANNE, PHD
PROFESSOR AND ASSOCIATE DEAN FOR RESEARCH
SCHOOL OF NURSING
EMORY UNIVERSITY
ATLANTA, GA 30322

DAVIS, CLAUDIA M, BSN, PHD *
ASSOCIATE PROFESSOR
CENTER FOR HEALTH DISPARITIES RESEARCH &
TRAINING
DEPARTMENT OF NURSING
COLLEGE OF NATURAL SCIENCES
CALIFORNIA STATE UNIVERSITY SAN BERNADINO
MORENO VALLEY, CA 92557

DE GROOT, MARY K, PHD *
ASSOCIATE PROFESSOR
DEPARTMENT OF MEDICINE
INDIANA UNIVERSITY SCHOOL OF MEDICINE
INDIANAPOLIS, IN 46202

DEATRICK, JANET A, RN, PHD, FAAN
PROFESSOR AND SHEARER ENDOWED TERM CHAIR
DEPARTMENT OF FAMILY AND COMMUNITY HEALTH
SCHOOL OF NURSING
UNIVERSITY OF PENNSYLVANIA
PHILADELPHIA, PA 19104

DEVON, HOLLI A, RN, FAHA, PHD, FAAN *
ASSOCIATE PROFESSOR
DEPARTMENT OF BIOBEHAVIORAL HEALTH SCIENCE
COLLEGE OF NURSING
UNIVERSITY OF ILLINOIS AT CHICAGO
CHICAGO, IL 60612

DOZIER, ANN MARIE, RN, PHD
PROFESSOR, INTERIM CHAIR
DEPARTMENT OF PUBLIC HEALTH SCIENCES
UNIVERSITY OF ROCHESTER
ROCHESTER, NY 14642

GIBSON, ROBERT WILLIAM, PHD *
PROFESSOR AND DIRECTOR OF RESEARCH
DEPARTMENT OF EMERGENCY MEDICINE
MEDICAL COLLEGE OF GEORGIA
GEORGIA REGENTS UNIVERSITY
AUGUSTA, GA 30912

GLICK, SUSAN B, MD *
ASSOCIATE PROFESSOR OF MEDICINE
DEPARTMENT OF INTERNAL MEDICINE
UNIVERSITY OF CHICAGO
CHICAGO, IL 60637

HODGE, FELICIA S, DRPH *
PROFESSOR
SCHOOL OF NURSING AND PUBLIC HEALTH
UNIVERISITY OF CALIFORNIA, LOS ANGELES
LOS ANGELES, CA 90095

HUPCEY, JUDITH E, RN, EDD, FAAN
PROFESSOR AND ASSOCIATE DEAN FOR GRADUATE
EDUCATION AND RESEARCH
COLLEGE OF NURSING
PENNSYLVANIA STATE UNIVERSITY
HERSHEY, PA 17033

JULION, WRENETHA A, RN, PHD
PROFESSOR
DEPARTMENT OF WOMEN, CHILDREN AND FAMILY
NURSING
COLLEGE OF NURSING
RUSH UNIVERSITY
CHICAGO, IL 60612

KEEFER, LAURIE A, PHD *
ASSOCIATE PROFESSOR
DEPARTMENT OF MEDICINE-GASTROENTERLOGY AND HEPATOL
PSYCHIATRY AND BEHAVIORAL SCIENCES
FEINBERG SCHOOL OF MEDICINE
NORTHWESTERN UNIVERSITY
CHICAGO, IL 60611

KELECHI, TERESA J, PHD
PROFESSOR
COLLEGE OF NURSING
MEDICAL UNIVERSITY OF SOUTH CAROLINA
CHARLESTON, SC 29425

KIM, MIYONG T, PHD, FAAN
PROFESSOR AND ASSOCIATE VICE PRESIDENT FOR COMMUNITY HEALTH ENGAGEMENT
DIVISION OF DIVERSITY COMMUNITY ENGAGEMENT
SCHOOL OF NURSING
UNIVERSITY OF TEXAS AT AUSTIN
AUSTIN, TX 78701

LAWLOR, MARY C, SCD *
PROFESSOR
DIVISION OF OCCUPATIONAL SCIENCE
OCCUPATIONAL THERAPY
UNIVERSITY OF SOUTHERN CALIFORNIA
LOS ANGELES, CA 90033

LEE, CHRISTOPHER SEAN, RN, PHD
ASSOCIATE PROFESSOR
SCHOOL OF NURSING
OREGON HEALTH AND SCIENCE UNIVERSITY
PORTLAND , OR 97239

MOORE, SHIRLEY M, RN, PHD, FAAN
PROFESSOR AND ASSOCIATE DEAN FOR RESEARCH
SCHOOL OF NURSING
CASE WESTERN RESERVE UNIVERSITY
CLEVELAND, OH 44106

NESS, KIRSTEN KIMBERLIE, PHD *
ASSOCIATE MEMBER
DEPARMENT OF EPIDEMIOLOGY AND CANCER CONTROL
ST. JUDE CHILDREN'S RESEARCH HOSPITAL
MEMPHIS, TN 38105

PAGE, GAYLE GIBONEY, RN, DNSC, FAAN
PROFESSOR AND INDEPENDENCE FOUNDATION CHAIR IN NURSING EDUCATION
SCHOOL OF NURSING
JOHNS HOPKINS UNIVERSITY
BALTIMORE, MD 21205

PATTERSON, RUTH E, PHD *
PROFESSOR OF FAMILY AND PREVENTIVE MEDICINE; PROGRAM LEADER, CANCER PREVENTION & CONTROL PROGRAM
DEPARTMENT OF FAMILY MEDICINE & PUBLIC HEALTH
MOORES CANCER CENTER
UNIVERSITY OF CALIFORNIA - SAN DIEGO
LA JOLLA, CA 92093

PORTER, STEPHEN C, MD *
ASSOCIATE PROFESSOR
DEPARTMENT OF PAEDIATRICS
EMERGENCY MEDICINE DIVISION
THE HOSPITAL FOR SICK CHILDREN
TORONTO, ONTARIO, M5G1X8
CANADA

RENN, CYNTHIA L, PHD *
ASSOCIATE PROFESSOR
SCHOOL OF NURSING
UNIVERSITY OF MARYLAND
BALTIMORE, MD 21201

ROSEN, MARC I, MD
ASSOCIATE PROFESSOR
DEPARTMENT OF PSYCHIATRY
YALE UNIVERSITY
NEW HAVEN, CT 06516

SENG, JULIA S, RN, PHD
ASSOCIATE PROFESSOR
DEPARTMENT OF OBSTETRICS AND GYNOCOLOGY
DEPARTMENT OF WOMEN'S STUDIES
SCHOOL OF NURSING
UNIVERSITY OF MICHIGAN
ANN ARBOR, MI 48109

SHEPPARD, VANESSA B, PHD
ASSOCIATE PROFESSOR
LOMBARDI COMPREHENSIVE CANCER CENTER
GEORGETOWN UNIVERSITY MEDICAL CENTER
WASHINGTON, DC 20007

SHERWOOD, PAULA R, RN, CNRN, PHD, FAAN
PROFESSOR, VICE CHAIR OF RESEARCH
DEPT. OF ACUTE & TERTIARY CARE, SCHOOL OF NURSING
DEPARTMENT OF NEUROLOGICAL SURGERY
SCHOOL OF MEDICINE
UNIVERSITY OF PITTSBURGH
PITTSBURGH, PA 15261

SOLE, MARY LOU, RN, PHD, FAAN
DEAN AND PROFESSOR
COLLEGE OF NURSING
UNIVERSITY OF CENTRAL FLORIDA
ORLANDO, FL 32826

STONE, PATRICIA W, RN, PHD, FAAN
CENTENNIAL PROFESSOR OF HEALTH POLICY
SCHOOL OF NURSING
COLUMBIA UNIVERSITY
NEW YORK, NY 10032

SULLIVAN, MARY C, PHD, FAAN *
PROFESSOR
COLLEGE OF NURSING
UNIVERSITY OF RHODE ISLAND
KINGSTON, RI 02881

SUMAN, OSCAR E, PHD
PROFESSOR
DEPARTMENT OF SURGERY
SCHOOL OF MEDICINE
UNIVERSITY OF TEXAS MEDICAL BRANCH
GALVESTON, TX 77550

SZALACHA, LAURA A, EDD *
PROFESSOR
DIRECTOR OF RESEARCH METHODS AND STATISTICS
COLLEGE OF NURSING
UNIVERSITY OF ARIZONA
TUCSON, AZ 85721

TANCREDI, DANIEL JOSEPH, PHD
ASSOCIATE PROFESSOR
DEPARTMENT OF PEDIATRICS
CENTER FOR HEALTHCARE POLICY AND RESEARCH
UNIVERSITY OF CALIFORNIA, DAVIS
SACRAMENTO, CA 95817

WANG, DONGWEN , PHD *
ASSOCIATE PROFESSOR
DEPARTMENT OF BIOSTATISTICS AND
MEDICAL INFORMATICS
SCHOOL OF MEDICINE AND DENTISTRY
UNIVERSITY OF ROCHESTER
ROCHESTER, NY 14642

WILKES, MICHAEL S, MD, PHD *
PROFESSOR
DEPARTMENT OF MEDICINE AND GLOBAL HEALTH
SCHOOL OF MEDICINE
UNIVERSITY OF CALIFORNIA, DAVIS
DAVIS, CA 95616

WILLIAMS, KAREN PATRICIA , PHD
PROFESSOR
DEPARTMENT OF OBSTETRICS, GYNECOLOGY
AND REPRODUCTIVE BIOLOGY
MICHIGAN STATE UNIVERSITY
EAST LANSING, MI 48824

MAIL REVIEWER(S)

BRENNAN, DANIEL C., MD
PROFESSOR OF MEDICINE
DEPARTMENT OF INTERNAL MEDICINE / RENAL DIVISION
DIRECTOR OF TRANSPLANT NEPHROLOGY
WASHINGTON UNIVERSITY SCHOOL OF MEDICINE
ST. LOUIS, MO 63110

CALHOUN, VINCE D, PHD
EXECUTIVE SCIENCE OFFICER AND DIRECTOR, IMAGE
ANALYTICS AND MR RESEARCH
DEPARTMENTS OF ELECTRICAL
AND COMPUTER ENGINEERING
UNIVERSITY OF NEW MEXICO
ALBUQUERQUE, NM 87131

CAPEL, BLANCHE , PHD
PROFESSOR OF CELL BIOLOGY
DEPARTMENT OF CELL BIOLOGY
DUKE UNIVERSITY MEDICAL CENTER
DURHAM, NC 27710

FAN, VINCENT S, MD
UNIVERSITY OF WASHINGTON
OFFICE OF SPONSORED PROGRAMS
1100 NE 45TH STREET, SUITE 300
SEATTLE, WA 98105

LORING, STEPHEN H, MD
SCIENTIFIC DIRECTOR
DEPARTMENT OF ANESTHESIA AND CRITICAL CARE
BETH ISRAEL DEACONESS MEDICAL CENTER
BOSTON, MA 02215

MATTHEWS, JUDITH T, PHD
RESEARCH ASSOCIATE PROFESSOR
DEPARTMENT HEALTH AND COMMUNITY SYSTEMS
SCHOOL OF NURSING
UNIVERSITY OF PITTSBURGH
PITTSBURGH, PA 15261

MUELLER, ELIZABETH ROSE
ASSOCIATE PROFESSOR
DEPARTMENT OF UROLOGY AND
OBSTERTRICS/GYNECOLOGY
LOYOLA UNIVERSITY MEDICAL CENTER
MAYWOOD, IL 60153

VARMA, ROHIT , MD
PROFESSOR AND CHAIR
DEPARTMENT OF OPHTHALMOLOGY
USC EYE INSTITUTE
UNIVERSITY OF SOUTHERN CALIFORNIA
LOS ANGELES, CA 90033

WU, LILY , MD, PHD
PROFESSOR
DEPARTMENT OF MOLECULAR AND MEDICAL
PHARMACOLOGY
DAVID GEFFEN SCHOOL OF MEDICINE AT
UNIVERSITY OF CALIFORNIA LOS ANGELES
LOS ANGELES, CA 90095

SCIENTIFIC REVIEW OFFICER

HARE, MARTHA L, PHD
SCIENTIFIC REVIEW OFFICER
CENTER FOR SCIENTIFIC REVIEW
NATIONAL INSTITUTES OF HEALTH
BETHESDA, MD 20892

EXTRAMURAL SUPPORT ASSISTANT

BARTLETT, VALERIE
EXTRAMURAL SUPPORT ASSISTANT
CENTER FOR SCIENTIFIC REVIEW
NATIONAL INSTITUTES OF HEALTH
BETHESDA, MD 20892

* Temporary Member. For grant applications, temporary members may participate in the entire meeting or may review only selected applications as needed.

Consultants are required to absent themselves from the room during the review of any application if their presence would constitute or appear to constitute a conflict of interest.
